# Tumor to normal single-cell mRNA comparisons reveal a pan-neuroblastoma cancer cell

**DOI:** 10.1126/sciadv.abd3311

**Published:** 2021-02-05

**Authors:** Gerda Kildisiute, Waleed M. Kholosy, Matthew D. Young, Kenny Roberts, Rasa Elmentaite, Sander R. van Hooff, Clarissa N. Pacyna, Eleonora Khabirova, Alice Piapi, Christine Thevanesan, Eva Bugallo-Blanco, Christina Burke, Lira Mamanova, Kaylee M. Keller, Karin P.S. Langenberg-Ververgaert, Philip Lijnzaad, Thanasis Margaritis, Frank C.P. Holstege, Michelle L. Tas, Marc H.W.A. Wijnen, Max M. van Noesel, Ignacio del Valle, Giuseppe Barone, Reinier van der Linden, Catriona Duncan, John Anderson, John C. Achermann, Muzlifah Haniffa, Sarah A. Teichmann, Dyanne Rampling, Neil J. Sebire, Xiaoling He, Ronald R. de Krijger, Roger A. Barker, Kerstin B. Meyer, Omer Bayraktar, Karin Straathof, Jan J. Molenaar, Sam Behjati

**Affiliations:** 1 Wellcome Sanger Institute, CB10 1SA Hinxton, UK.; 2 Princess Máxima Center for Pediatric Oncology, Heidelberglaan 25, 3584 CS Utrecht, Netherlands.; 3 UCL Great Ormond Street Institute of Child Health, WC1N 1EH London, UK.; 4 Great Ormond Street Hospital for Children (GOSH), NHS Foundation Trust, NIHR Great Ormond Street Hospital Biomedical Research Centre, WC1N 3JH London, UK.; 5 Hubrecht Institute, KNAW, 3584 CT Utrecht, Netherlands.; 6 Institute of Cellular Medicine, Newcastle University, NE2 4HH Newcastle upon Tyne, UK.; 7 Department of Dermatology and NIHR Newcastle Biomedical Research Centre, Newcastle Hospitals, NHS Foundation Trust, NE2 4LP Newcastle upon Tyne, UK.; 8 MRC-WT Cambridge Stem Cell Institute, University of Cambridge, CB2 0QQ Cambridge, UK.; 9 Department of Clinical Neurosciences, University of Cambridge, CB2 0QQ Cambridge, UK.; 10 Department of Pathology, University Medical Center Utrecht, Heidelberglaan 100, 3584 CX Utrecht, Netherlands.; 11 Cambridge University Hospitals NHS Foundation Trust, CB2 0QQ Cambridge, UK.; 12 Department of Paediatrics, University of Cambridge, CB2 0QQ Cambridge, UK.

## Abstract

Neuroblastoma is a childhood cancer that resembles developmental stages of the neural crest. It is not established what developmental processes neuroblastoma cancer cells represent. Here, we sought to reveal the phenotype of neuroblastoma cancer cells by comparing cancer (*n* = 19,723) with normal fetal adrenal single-cell transcriptomes (*n* = 57,972). Our principal finding was that the neuroblastoma cancer cell resembled fetal sympathoblasts, but no other fetal adrenal cell type. The sympathoblastic state was a universal feature of neuroblastoma cells, transcending cell cluster diversity, individual patients, and clinical phenotypes. We substantiated our findings in 650 neuroblastoma bulk transcriptomes and by integrating canonical features of the neuroblastoma genome with transcriptional signals. Overall, our observations indicate that a pan-neuroblastoma cancer cell state exists, which may be attractive for novel immunotherapeutic and targeted avenues.

## Teaser

Comparisons of neuroblastoma cells and relevant normal adrenal cells reveal a uniform cell state that characterizes neuroblastoma.

## INTRODUCTION

Neuroblastoma is a childhood cancer that exhibits a diverse pattern of disease (*1*), from spontaneously resolving tumors to a highly aggressive cancer. Neuroblastoma arises from aberrant differentiation of the neural crest, mostly within the fetal adrenal medulla, via the sympathetic lineage. Different stages of adrenal medullary cell differentiation have been implicated as the cell of origin of neuroblastoma (*2*–*4*) and proposed to underlie the diversity of clinical phenotypes (*2*, *5*).

Advances in single-cell transcriptomics have enabled the direct comparison of cancer and normal reference cells. Applied to childhood cancer, such analyses have revealed specific cell types or developmental processes that tumors adopt and adapt (*6*). Here, we sought to establish, through cancer to normal single-cell mRNA comparisons, which developmental processes and cell types neuroblastoma recapitulates and correlate our findings with disease phenotypes.

## RESULTS

### Building a reference of human fetal medullary cells

**Fig. 1:**
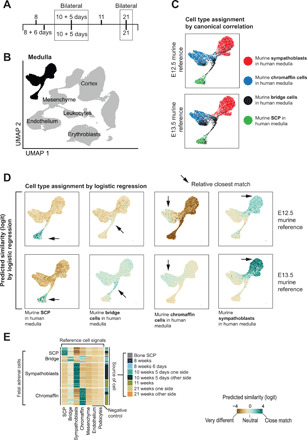
Single-cell reference data from seven human fetal adrenal glands. (**A**) Gestational age of fetal adrenal glands. (**B**) UMAP (Uniform Manifold Approximation and Projection) representation of 57,972 fetal adrenal gland cells. Medullary cells (*n* = 6451) are highlighted in black. (**C**) Cell type assignment by canonical correlation analysis as implemented in the TransferData function in Seurat. Murine medullary cell types, as defined by Furlan *et al*. (*8*), from two developmental ages [embryonic day (E) 12.5 and E13.5] were transferred to human medullary cells [black cell population in (A)] to identify human correlates (as per color legend). (**D**) Cell type assignment by logistic regression comparing murine medullary cell types, as defined by Furlan *et al*. (*8*) from two developmental ages (E12.5 and E13.5) with our human data [black cell population in (A)]. Colors represent the similarity score (logit scale): 0 default, positive (green) indicating strong similarity and negative (brown) strong dissimilarity. (**E**) Comparison of medullary cells from different gestational ages. Similarity scores are shown by color, comparing the reference week 10, day 5 adrenal gland, and extrinsic control (*x* axis) to medullary cell types across all time points (*y* axis). The annotation track to the right of the heatmap shows the sample each cell belongs to.

In the first instance, we built a normal cell reference for our analyses by quantitatively defining transcription of cells underpinning human adrenal development. We undertook single-cell mRNA sequencing (10x Genomics Chromium platform) of seven adrenal glands obtained from first and second trimester human fetuses (Fig. 1A and table S1). To verify technical and biological replicability, we included bilateral adrenal glands in two cases. Following stringent data filtering, including removal of doublets and ambient mRNAs, we obtained count tables of gene expression from a total of 57,972 cells. These cells could broadly be divided into cortical, medullary, and mesenchymal cells and leukocytes (Fig. 1B and fig. S1A) using canonical human adrenal markers (fig. S1B and table S2). We focused our analyses on medullary cells (*n* = 6451), from which most cases of neuroblastoma arise (*7*). The absolute and relative number of medullary cells varied across medullas, likely reflecting technical and biological differences, such as the relative proportion of cortex to medulla (table S1).

Recent single-cell analyses of murine fetal adrenal glands have provided transcriptional definitions of four principal medullary cell types (*8*–*10*): Schwann cell precursor (SCP), bridge cells, chromaffin cells, and sympathoblasts. To search for these cell types within our data, we used two orthogonal unbiased methods (logistic regression and canonical correlation analysis) to quantitatively map murine reference signals onto our human data. Unlike manual annotation of cells by a selection of (biased) marker genes, these approaches avoid the risk of misclassifying cells when interspecies differences on individual markers exist. Accordingly, we identified the human correlates of the four medullary cell types (Fig. 1, C and D). Of particular note were the neural crest–like SCP cells, identified previously in murine (*8*), but not human, fetal tissues. We validated these cells by single-molecule fluorescence in situ hybridization (FISH) of SCP defining markers (*SOX10*, *ERBB3*, *MPZ*, and *PLP1*) in the fetal medulla and other human fetal organs—fetal gut, eye, skin, and bone (fig. S2). Comparing the four medullary cell types across different adrenals did not reveal notable differences in their overall transcriptional “identity” (Fig. 1E), although nuances in transcription may exist across gestational ages (table S3). We therefore merged cell signals from different adrenals to form the reference for cancer-to-normal cell comparisons and for additional analyses on fetal medullary cells, including trajectory analyses and comparison to murine data (figs. S1C and S3).

### Identification of cancer cells in neuroblastoma

**Fig. 2:**
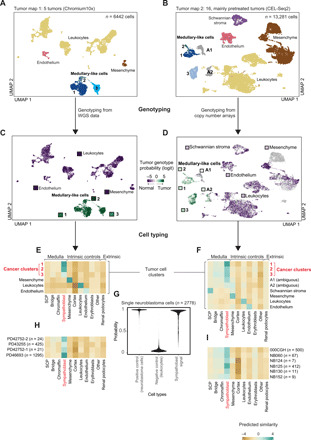
Neuroblastoma single-cell transcriptomes. (**A** and **B**) UMAP representation of 6442 (A) and 13,281 (B) neuroblastoma cells. Colors and labels indicate different cell types defined using marker gene expression. (**C** and **D**) The same UMAP representation as in (A) and (B), but here, color represents posterior probability of the cancer genotype calculated for each cell. The square next to each cluster title shows the average posterior probability for that cluster. Scores are given on a logit scale, with 0 indicating no information, positive (green) values evidence for the tumor genotype, and negative (purple) evidence for the normal genotype. (**E** and **F**) Similarity scores (logistic regression and logit scale) of the reference fetal adrenal gland (*x* axis) to neuroblastoma cells (*y* axis), containing intrinsic (noncancer cells) and extrinsic (kidney podocytes) control cell population. (**G**) Tumor to normal similarity score, represented as posterior probability, comparing neuroblastoma cells (positive control), leukocytes (negative control), and sympathoblasts to neuroblastoma cells. The similarity of tumor cells to sympathoblasts is comparable to the similarity of tumor cells to themselves. (**H** and **I**) Similarity of cells from the cancer clusters in (E) and (F) grouped by patient. Numbers in parentheses indicate the total number of cells in cancer clusters in (E) and (F). Only patients with at least 10 tumor cells or at least one tumor cell with validated genotype were included.

We next sought to establish the relationship between neuroblastoma cells and human fetal medullary development. We generated single-cell mRNA readouts from 21 fresh neuroblastoma specimens from two different centers using two platforms (Chromium 10x and CEL-Seq2). We analyzed data from these different platforms and centers independently to ascertain technical and biological replicability of our findings. We obtained tissue from untreated patients at diagnosis (*n* = 4) or following treatment with cytotoxic agents at resection (*n* = 17) (table S1). As pretreated neuroblastomas tend to be largely necrotic at surgical resection, we guided sampling to viable tumor areas through preoperative metabolic cross-sectional imaging in these specimens (meta-iodobenzylguanidine scan), coupled with morphological assessment of frozen sections in 15 of 17 pretreated cases. Our study cohort represented the three principal prognostic categories of neuroblastoma: low-risk (*n* = 3), intermediate-risk (*n* = 5), and high-risk (*n* = 13) cases. In total, we obtained 19,723 cells, with variable contribution from each tumor (Fig. 2, A and B, and table S1), which segregated into four main cell types: leukocytes (*n* = 10,593), mesenchymal cells (*n* = 4227), cells bearing markers of Schwannian stroma (*n* = 665), and putative tumor cells exhibiting adrenal medullary–like features (*n* = 3396).

The first challenge was to identify bona fide cancer cells among tumor-derived cells. Marker-based cell typing alone was of limited value, as controversy exists as to which cell types in neuroblastoma represent cancer cells. Although there is a general consensus that neuroblastoma cancer cells exhibit medullary-like features, it has also been suggested that interstitial (mesenchymal) and Schwannian stroma cells, commonly found in neuroblastoma, may be cancerous (*11*–*13*). We therefore used somatic copy number changes to identify cancer cells by interrogating each cell’s mRNA sequence for evidence of the somatic copy number changes underpinning each tumor. Extending a previously developed method (*6*), we integrated single-cell tumor RNA information with single-nucleotide polymorphism (SNP) arrays or whole-genome sequencing of the tumor DNA (table S4). This allowed us to assess the allelic imbalance of SNPs in each cell’s mRNA sequence across patient-specific copy number segments. This analysis revealed that in our cohort only adrenal medullary–like cells, but not interstitial or Schwannian stroma cells, were cancerous (Fig. 2, C and D).

### Comparison of cancer to normal cells at single-cell resolution

Next, we investigated which stage of adrenal medullary development cancer cells recapitulate by performing cancer to normal cell comparisons using two independent methods. We found that neuroblastoma cancer cells did not recapitulate adrenal development but had assumed the state of sympathoblasts (Fig. 2, E to G, and fig. S4A). Although cancer cells formed discrete clusters within and across patients (Fig. 2, A and B), the cancer to normal cell comparison resolved this diversity into a common sympathoblast-like phenotype (Fig. 2, H and I). The sympathoblast state was present in all patients (Fig. 2, H and I).

**Fig. 3:**
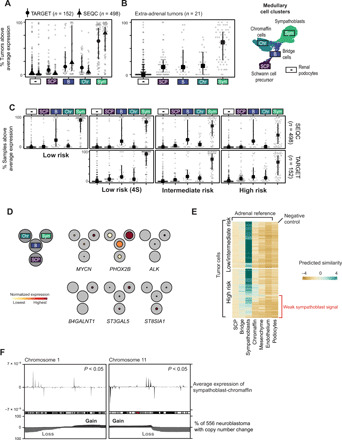
Validation in clinically annotated neuroblastoma cohorts. (**A**) Fraction of bulk neuroblastoma samples in TARGET (Therapeutically Applicable Research To Generate Effective Treatments) cohort and SEQC (Sequencing Quality Control) with above average marker expression. Dataset/cell type is indicated by points/*x* axis labels (see UMAP on right), lines indicate interquartile ranges, symbols indicate the median. (**B**) As in (A), but limited to tumors sampled outside the adrenal gland. (**C**) As in (A), but with samples split by clinical risk group (horizontal facets) and datasets (vertical facets). (**D**) Fetal medulla expression of GD2 synthesis genes, principal somatic, and germline predisposition genes. Black circles represent cell populations in (B), with average expression (normalized for each gene)/fraction of expressing cells indicated by color/colored circle radius. Further genes shown in fig. S5. (**E**) Similarity scores (logistic regression and logit scale) comparing fetal adrenal (*x* axis) to neuroblastoma cells (*y* axis) and extrinsic control (kidney podocytes). In high-risk tumor cells, a subset of cells exhibit a weakened sympathoblast signal, indicated by the red label. (**F**) Average expression difference between sympathoblastic and chromaffin cells versus of genomic position on chromosomes 1 and 11 (top). Positive (negative) values indicate higher expression in sympathoblasts (chromaffin) cells. The bottom shows fraction of 556 neuroblastomas with copy number gain (black) or loss (gray).

To validate that neuroblastoma cancer cells principally resemble sympathoblasts, we interrogated neuroblastoma bulk transcriptomes. We curated gene expression profiles of 650 neuroblastomas from two different clinically annotated cohorts that represent the entire clinical spectrum of neuroblastoma [Therapeutically Applicable Research To Generate Effective Treatments (TARGET) (*n* = 152) (*14*) and Sequencing Quality Control (SEQC) (*n* = 498) (*15*) cohorts]. We probed these expression data for transcripts that define the four populations of the normal human fetal medulla: SCPs, bridge cells, sympathoblasts, and chromaffin cells (fig. S1B). Across cohorts, we found a clear signal of sympathoblast mRNAs (Fig. 3A), thus verifying the sympathoblastic state of neuroblastoma. The sympathoblast signal in bulk transcriptomes was reproduced by using murine reference data (fig. S4C). Neuroblastomas that arose outside the adrenal gland also exhibited sympathoblast signals (Fig. 3B). The sympathoblast signal was present across the principal risk groups of neuroblastoma, suggesting that it transcends the clinical diversity of neuroblastoma (Fig. 3C).

### Features of the neuroblastoma genome corroborate the sympathoblast state

To further corroborate the transcriptional evidence that neuroblastoma cells resemble sympathoblasts, we overlaid features of the neuroblastoma cancer genome with transcriptional developmental pathways. We first analyzed how somatic cancer (*14*) and germline predisposition (*16*) genes of neuroblastoma are used by fetal medullary cells. We found that the majority of these genes were most highly expressed in sympathoblasts (Fig. 3D and fig. S5A), in particular, the two most common neuroblastoma predisposition genes, *ALK* and *PHOX2B*, and the cancer gene *MYCN*, somatic amplification of which is a key adverse marker used clinically for neuroblastoma risk stratification. Thus, the same genes that operate as cancer genes in neuroblastoma are used in normal development predominantly by sympathoblasts. Similarly, genes encoding the synthases of disialoganglioside (GD2), which is a near-ubiquitous marker of neuroblastoma that is therapeutically targeted by antibody treatment (*17*), were predominantly expressed in sympathoblasts (Fig. 3D). We note that the expression of the enzyme catalyzing the final step in GD2 synthesis, *B4GALNT1*, was confined to sympathoblasts, whereas genes further upstream in the synthesis pathway, *ST3GAL5* and *ST8SIA1*, showed a less restricted pattern of expression within the medulla.

A variety of copy number changes have been described in neuroblastoma, the most recurrent and pertinent of which occur on chromosomes 1, 11, and 17 (*18*). The presence of these copy number changes carries fundamental prognostic significance and determines treatment intensity in most clinical contexts and treatment protocols. Given the sympathoblastic state of neuroblastoma, it seemed conceivable that somatic copy number changes may affect the expression of genes that define sympathoblasts. We were able to directly test this hypothesis. For example, we compared gene expression of cancer cells that harbor loss of chromosomes 1 and 11 with cancer cells that do not carry these changes (table S5). We found that the resulting differentially expressed genes were significantly enriched for high confidence sympathoblast marker genes (tf-idf > 1, 283 of 329 markers; *P* < 0.0001, hypergeometric test] (table S6). Furthermore, most of these genes (254 of 329) exhibited lower expression in cells with chromosome 1 and 11 loss, even if those genes did not reside on chromosome 1 or 11. Following on from this, we correlated genomic regions of recurrent chromosome 1 or 11 loss with the genomic localization of gene expression in fetal medullary cells. The localization of the boundaries of altered copy number segments is associated with gene expression in human cancer (*19*). Thus, the genomic position of these boundaries may encode information about the expression profile of the cancer cell of origin. We found regions of sympathoblast-specific gene expression that statistically significantly overlapped with the most common boundaries of chromosome 11q loss (*P* < 0.05, permutation test) (Fig. 3F and fig. S5B) and the most recurrent region of chromosome 1p loss (*P* < 0.05, permutation test) (Fig. 3F and fig. S5C), thus further corroborating the sympathoblast state of neuroblastoma.

### Differences between low- and high-risk tumors

The measurement of medullary cell signals in bulk transcriptomes indicated that, compared to lower-risk tumors, the sympathoblast signal was less pronounced in high-risk tumors in both cohorts (Fig. 3C). This weakened sympathoblast signal may be driven by differences in tissue composition or by distinct properties of cancer cells themselves. We reanalyzed the sympathoblast signal in single cancer cells, this time subdividing neuroblastoma cells by whether they originated from low- or high-risk tumors. We found that some high-risk, but not low-risk, cells exhibited a substantially decreased sympathoblast signal (Fig. 3E). This difference could not be accounted for by technical variation (fig. S4B). Differential gene expression between the two groups of high-risk cells showed that the weakened sympathoblast signal was underpinned by, among other mRNAs, transcripts defining neuronal and neuroendocrine lineages (table S7). In aggregate, these findings indicate that a subtype of high-risk cell may exist, which is characterized by a fainter sympathoblast signal.

### Utilization of fetal transcripts in neuroblastoma

**Fig. 4:**
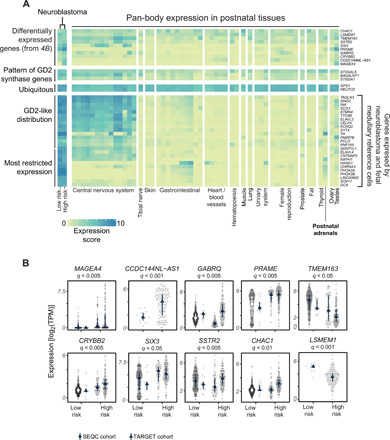
Fetal neuroblastoma genes and prognostically significant neuroblastoma mRNAs. (**A**) A heatmap showing the expression of GD2 synthesis genes, genes from (B), and genes used by both neuroblastoma cells and the fetal medulla (*y* axis) in bulk transcriptomes of normal tissues (GTEX) and high-/low-risk neuroblastomas from TARGET. The color indicates log_2_ of the average gene expression (TPM) for the corresponding gene and sample set. (**B**) Genes differentially expressed between neuroblastoma tumor cells and fetal medulla that exhibit significant risk group dependence after controlling for MYCN status and age (negative binomial test, log_2_FC > 1, FDR < 0.05) in TARGET dataset. For the gene listed above each panel, dots indicate expression [log_2_(TPM)] in individual samples, lines indicate the interquartile range, and symbols indicate the median for SEQC and TARGET datasets. Two genes (*LSMEM1* and *CCDC144NL-AS1*) are not present in annotation used by SEQC.

As neuroblastoma cells use transcripts of fetal medullary cells, it is conceivable that the expression of some of these genes is relatively restricted to the fetal period and may thus represent formidable therapeutic targets. The precedent for this concept is the disialoganglioside, GD2, which is widely expressed by neuroblastoma cells and some fetal lineages including sympathoblasts (Fig. 4A). In postnatal tissues, however, expression of key genes encoding GD2 synthases is most confined to the central and peripheral nervous systems (*20*). Therapeutic antibodies targeting GD2 are deployed in most treatment protocols of high-risk neuroblastoma. Although pain, probably mediated by expression in peripheral nerves, is a common adverse effect, significant central nervous system (CNS) toxicity is rare. To identify transcripts with a similar distribution to GD2 synthases, we searched for mRNAs used by single neuroblastoma and fetal medullary cells (hereinafter referred to as fetal cancer transcript). We then assessed their distribution across pan-body postnatal tissues [from Genotype-Tissue Expression (GTEx) data (*21*)] while validating their expression in neuroblastoma bulk transcriptomes. These analyses revealed two patterns of fetal cancer gene expression across postnatal tissues. One pattern followed the distribution of mRNAs encoding GD2, whereby expression of fetal cancer transcripts was predominantly expressed in neuroblastoma and in CNS tissues. Some genes, including those pertaining to catecholamine synthesis, exhibited an even more restricted expression pattern. Together, these analyses reveal a number of fetal cancer genes that may lend themselves as targets in neuroblastoma (table S8).

### Differences between neuroblastoma and normal fetal medullary cells

Having established the similarities between neuroblastoma cancer cells and fetal medullary development, we now looked for differences. Previous bulk mRNA analyses have described gene expression changes that characterize neuroblastoma tissues (*3*, *22*). Our ability to directly compare cancer cells with their normal counterpart, fetal medullary cells, enabled us to distil the transcriptional essence of neuroblastoma cells, which comprises 91 differentially expressed genes (tf-idf > 0.85; table S9). To verify, and to correlate, these findings with disease phenotypes, we measured mRNA levels of these differentially expressed genes in bulk neuroblastoma transcriptomes and ascertained that a significant number of them had above average gene expression (*n* = 37 of 89; *P* < 0.001, hypergeometric test; fig. S6). The expression of 10 of these varied significantly by clinical risk group, even after controlling for key determinants of clinical risk, age, and *MYCN* amplification (negative binomial generalized linear model) (Fig. 4B). In addition to known markers of clinical risk (e.g., *PRAME*), we identified markers that have not previously been described and could, in theory, be used to improve risk stratification in clinical practice in the future. Biologically, the transcription factor gene *SIX3* may be one of the most interesting findings among differentially expressed genes, representing an embryonal gene that normally regulates forebrain and eye development (*23*).

## DISCUSSION

The principal finding of our investigation was that the neuroblastoma cancer cell represented an aberrant fetal sympathoblast. Our expectation might have been that neuroblastoma exhibits a cellular hierarchy adopted from adrenal development, similar to, for example, the childhood kidney cancer, Wilms tumor, recapitulating fetal nephrogenesis. However, neither our direct analyses of single cancer cells nor indirect insights from bulk cancer transcriptomes would corroborate such a hypothesis.

We identified cancer cells by directly assessing SNP ratios in regions of cancer defining copy number changes rather than inferring copy number from gene expression levels. Accordingly, we did not find evidence that cells other than sympathoblast-like cells were malignant. Therefore, our findings suggest that the sympathoblastic state predominates across the entire spectrum of neuroblastoma. The transcriptional similarity between neuroblastoma cancer cells and sympathoblasts may indicate that neuroblastoma directly derives from sympathoblasts. However, it is also conceivable that neuroblastoma arises from other cell states of the neural crest lineage that assume the transcriptional state of sympathoblasts upon malignant transformation.

The neuroblastoma transcriptome has been studied extensively from bulk tissues, revealing a plethora of potential targets that have been evaluated over past decades. Nevertheless, leveraging the resolution of single cells to perform precise comparison of cell types, we identified additional potential targets in neuroblastoma including fetal genes that are used by neuroblastoma cells with a restricted expression profile in postnatal tissues. Furthermore, direct comparison of cancer and fetal medullary cells revealed neuroblastoma genes that correlate with outcome in two independent validation cohorts, which may prove useful for refined risk stratification.

Overall, our findings reveal an unexpected homogeneity in the cellular identity of neuroblastoma. Although future, larger-scale analyses may define the nuances of this signal and perhaps identify rare cancer cell types, our study would suggest that the principal normal cell correlate of neuroblastoma is the human fetal sympathoblast.

## MATERIALS AND METHODS

### Ethics statement

Informed consent for research was obtained from participants (or their carers). Studies underlying this paper have received appropriate approval by ethics review boards as per national legislation. Dutch tumor samples were obtained through an institutionally approved research study. U.K. tumor samples were collected under the following studies: National Health Service (NHS) National Research Ethics Service reference 16/EE/0394 (tumor samples) and NHS National Research Ethics Service reference 96/085 (fetal tissues). Additional fetal tissue was provided by the Joint Medical Research Council (MRC)/Wellcome Trust–funded (grant #099175/Z/12/Z) Human Developmental Biology Resource (HDBR; www.hdbr.org) (*10*), with appropriate maternal written consent and approval from the Newcastle and North Tyneside NHS Health Authority Joint Ethics Committee. HDBR is regulated by the U.K. Human Tissue Authority (HTA; www.hta.gov.uk) and operates in accordance with the relevant HTA Codes of Practice.

### Fetal adrenal tissue processing

Tissue was minced with a scalpel and then placed in a 15-ml falcon tube filled with 5 ml of digestion solution [5 ml; dilute Liberase TH stock solution (one vial of 5 mg of Liberase TH (Sigma-Aldrich, 5401135001) powder in 2 ml of 1× phosphate-buffered saline (PBS) with a final concentration of 2.5 mg/ml] in 1× PBS (100 μl of enzyme stock + 4.9 ml of 1× PBS). Tissue was incubated at 37°C for 30 min (water bath) and mixed in 1-ml pipette after 15 min to facilitate the digestion. Five milliliters of STOP solution [40 ml; 2% fetal bovine serum (FBS) in 1× PBS (40 ml of 1× PBS + 800 μl of FBS)] was added. The mixture was filtered through a 30-μm strainer and spun down at 750*g* for 5 min at 4°C. If the pellet was red, 1 ml of 1× red blood cell (RBC) (00-4300-54) lysis buffer was added and incubated for 3 min at room temperature. Ten milliliters of STOP solution was added, and cells were gently mixed to wash and then centrifuged at 750*g* for 5 min at 4°C. Pellets were resuspended into appropriate amount of 1× PBS or STOP solution, counted, and processed on 10x Chromium platform.

### Neuroblastoma tissue processing—GOSH (Great Ormond Street Hospital) pipeline

Surplus tumor tissue obtained at diagnostic biopsy or tumor resection was processed immediately after receipt in the histopathology laboratory (<1 hour after interventional radiology/surgical procedure). Tissue was minced using a scalpel and then incubated in RPMI 1640, supplemented with 10% fetal calf serum, 1% l-glutamine, and 1% penicillin/streptomycin, with collagenase IV (1.6 mg/ml; catalog no. 11410982; MP Biomedicals), for 30 min at 37°C, inverting the tube every 10 min. The digested tissue was passed through a 70-μm filter and incubated in 1× RBC lysis buffer (catalog no. 420301; BioLegend) for 10 min at room temperature. The obtained single-cell suspension was used for downstream processing. Part of the single-cell suspension was depleted of CD45^+^ cells to enrich for tumor cells using a CD45 MicroBeads kit (catalog no. 130-045-801; Miltenyi Biotec), following the manufacturer’s protocol. Both CD45 nondepleted and CD45-depleted single-cell suspensions were depleted of dead cells using a Dead Cell Removal kit (catalog no. 130-090-101; Miltenyi Biotec), following the manufacturer’s protocol. Obtained viable single-cell suspensions (CD45 depleted: two channels and CD45 nondepleted: one channel) were processed on the 10x Chromium platform.

### Neuroblastoma tissue processing—PMC pipeline

Tumor tissue was collected after obtaining informed consent of the parents of neuroblastoma patients and after the study was approved by the ethical committee at the Prinses Máxima Centrum (PMC). Metaiodobenzylguanidine (MIBG)–positive neuroblastoma samples were included if they were diagnosed with histologically proven viable tumor cells. Most samples were debulking neuroblastomas (patients were treated with debulking-type surgical procedure), and preoperative radiation and chemotherapy had been given to the patients (table S1). We extensively optimized the workup protocols, and samples were gently dissociated to the single-cell level and sorted by flow cytometry. Single-cell suspensions were subsequently subjected to single-cell RNA sequencing (RNA-seq) using the CEL-Seq2 platform. In brief, freshly resected human neuroblastoma specimens were collected and processed immediately following histological confirmation of the presence of viable tumor tissue. Tumor tissue was minced into 3- to 4-mm pieces and digested with collagenase types I, II, and IV (2 mg/ml) in the optimized medium at 37°C with agitation for 2 hours. The resulting suspension containing small tumor fragments was passed through a 70-μm cells strainer and washed with a cold organoid medium. Half of the tumor fragments were used to generate tumor organoids, while the other half were fragments that were further dissociated with NeuroCult kit until the single-cell suspension was obtained. Cells were washed with cold organoid medium and stained with 7-aminoactinomycin D (7AAD) or 4′,6-diamidino-2-phenylindole (DAPI) to enrich for live cells, while others were stained with fluorescein isothiocyanate (FITC)–GD2 or phycoerythrin (PE)–GD2 antibodies to enrich for tumor and with PE-CD3 antibodies to enrich for T cells and to exclude erythrocytes. Cells were stored on ice until sorting using FACSJazz or FACSAria II. Single-live cells were sorted into 384-well hard-shell plates with 10 μl of mineral oil, 50 nl of reverse transcription primers, deoxynucleotide triphosphates (dNTPs), and synthetic mRNA Spike-Ins and immediately spun down, snap-frozen on dry ice, and stored at −80°C to proceed with the total transcriptome amplification, library preparation, and sequencing.

### 10x library preparation and sequencing

The concentration of single-cell suspensions was manually counted using a hemocytometer and adjusted to 1000 cells/μl or counted by flow cytometry. Cells were loaded according to the standard protocol of the Chromium Single Cell 3′ Kit (v2 and v3 chemistry). All the following steps were performed according to the standard manufacturer’s protocol. We used one lane of Illumina HiSeq 4000 per 10x chip position.

### CEL-Seq2 library preparation and sequencing

All samples that were processed proceeded with the total transcriptome amplification, library preparation, and sequencing into Illumina sequencing libraries as previously described (*24*). Paired-end 2 × 75–base pair (bp) sequencing read length was used to sequence the prepared libraries using an Illumina NextSeq sequencer.

### Bulk DNA processing of GOSH samples

DNA was extracted from fresh-frozen tissue. Peripheral blood DNA was used as a matched normal. Short insert (500 bp) genomic libraries were constructed, flow cells were prepared, and 150-bp paired-end sequencing clusters were generated on the Illumina HiSeq X platform according to Illumina no-PCR (polymerase chain reaction) library protocols (*25*). The average sequence coverage was 43× and 42× for tumor and matched peripheral blood samples, respectively.

### Bulk DNA processing of PMC samples

DNA was extracted from fresh tissue via the AllPrep DNA/RNA/Protein Mini Kit (QIAGEN) according to standard protocol.

### Bulk DNA alignment

DNA sequencing reads were aligned to the GRCh 37d5 reference genome using the Burrows-Wheeler transform (BWA-MEM) (*26*). Sequencing depth at each base was assessed using bedtools coverage v2.24.0.

### Single-molecule FISH

Fresh tissue samples were embedded in OCT (Optimal cutting temperature compound) and frozen at −80°C on an isopentane dry ice slurry. Cryosections were cut at a thickness of 16 μm using a Leica CM3050 S cryostat and placed onto SuperFrost Plus slides (VWR). Before staining, tissue sections were postfixed in 4% paraformaldehyde in PBS for 15 min at 4°C and then dehydrated through a series of 50, 70, 100, and 100% ethanol for 5 min each. Tissue sections were then processed using Leica BOND RX to automate staining with the RNAscope Multiplex Fluorescent Reagent Kit v2 Assay and RNAscope 4-plex Ancillary Kit for Multiplex Fluorescent Reagent Kit v2 (Advanced Cell Diagnostics, Bio-Techne), according to the manufacturers’ instructions. Automated processing included pretreatment with protease IV for 30 min, but no heat treatment. Tyramide signal amplification with Opal 520, Opal 570, and Opal 650 (Akoya Biosciences) was used to develop three probe channels. The fourth was developed using trichostatin A (TSA)–biotin (TSA Plus Biotin Kit; PerkinElmer) and streptavidin-conjugated Atto 425 (Sigma-Aldrich). Stained sections were imaged with the PerkinElmer Opera Phenix High Content Screening System in confocal mode with a 1-μm z-step size, using 20× [numerical aperture (NA) 0.16, 0.299 μm per pixel] and 40× (NA 1.1, 0.149 μm per pixel) water-immersion objectives.

### Copy number detection from DNA data

The ascatNGS algorithm (*27*) (v4.0.1) was used to estimate tumor purity and ploidy and to construct copy number profiles before running the Battenberg algorithm (v2.2.5) (github.com/cancerit/cgpBattenberg) to allow for tumor subclonality in bulk DNA sequencing data. For DNA data measured with Illumina SNP arrays, copy number segments were defined by manual inspection of logR and B allele frequency data.

### Mapping and quantification of single-cell RNAseq—10x

Single-cell RNA-seq data were mapped, and counts of molecules per barcode were quantified using the 10x software package cellranger (versions 2.0.2 and 3.0.2) to map sequencing data to version 2.1.0 of the build of the GRCh38 reference genome supplied by 10x.

### Mapping and quantification of single-cell RNA-seq—CEL-Seq2

Sharq preprocessing and the QC pipeline were applied to process the single-cell RNA-seq data as described (*28*). Read mapping was done using STAR version 2.6.1 on the hg38 Patch 10 human genome. The featureCounts function of the subread package (version 1.5.2) was used to assign reads based on GENCODE version 26.

### QC of fetal adrenal single-cell data

Ambient mRNA contamination was removed from each channel individually using SoupX (*29*). Cleaned count data were then normalized and scaled to have a mean of 0 and an SD of 1 for each gene, principal components analysis was performed using highly variable genes, and data were grouped into clusters using a community detection finding algorithm taking the first 75 principal components as inputs and a resolution parameter of 10. This was performed using the Seurat package in R (*30*, *31*). The clustering parameter was deliberately set to this extremely high value to produce a very large number of small clusters.

Following clustering, we designated each cell as either passing or failing a series of QC metrics. We marked as failing QC any cell with >30% expression due to mitochondrial genes, fewer than 300 genes detected, fewer than 1000 UMIs (Unique molecular identifiers) detected, or a doublet score greater than 0.2 as determined by Scrublet (*32*). In addition to excluding these individual cells, we also marked as failing QC any cell belonging to a high-resolution cluster containing more than 50% of cells that fail QC due to one of the above filters. Last, we excluded from all further analysis any cell marked as QC failed for any of the above reasons (fig. S7A).

Next, we calculated an S and G_2_-M phase score for each cell using Seurat (*30*, *31*) and excluded any cell where either score was greater than 0. This removed any cells that showed evidence of being in a phase of the cell cycle other than G_0_-G_1_. We excluded these cells from our reference map, as cells in the S/G_2_-M phase tend to cluster together based on their phase of cell cycle and not their underlying cell type, reducing the utility of the data as a reference map of cell types present in the developing adrenal gland.

As before, data were normalized by sequencing depth, scaled to 10,000 counts, and log-transformed, and genes were scaled to have a mean of 0 and an SD of 1. Principal components analysis was performed on the scaled data using the most highly variable genes. We selected the number of principal components to use for clustering and visualization to minimize the molecular cross-validated error (https://github.com/constantAmateur/MCVR) (*33*). Using these principal components, we calculated a uniform manifold approximation and projection (UMAP) (*34*) two-dimensional representation of the data for visualization and calculated clusters using a *k*-nearest neighbors algorithm with resolution parameter set to 1.

### QC of 10x tumor data

We excluded nuclear mitochondrial genes, heat shock proteins, and ribosomal genes from our analysis. To filter lower-quality cells, we removed any cells that had greater than 20% expression originating from mitochondrial genes, expressed fewer than 300 distinct genes, or contained fewer than 1000 UMIs (fig. S7B). Data were normalized by sequencing depth, scaled to 10,000 counts, and log-transformed, and genes were scaled to have a mean of 0 and an SD of 1 using the Seurat “NormalizeData” function. Principal components analysis was performed on the scaled data using the 2000 most variable genes. Using 75 first principal components, we calculated a UMAP (*34*) two-dimensional representation of the data for visualization and calculated clusters using a community detection algorithm with resolution parameter set to 1. We performed these steps using the Seurat package in R (*30*, *31*).

### QC of CEL-Seq2 tumor data

We removed RNA spike-ins and genes that were not present in the 10x gene list. We excluded nuclear mitochondrial genes, heat shock proteins, and ribosomal genes from our analysis. To filter lower-quality cells, we removed any cells that had greater than 20% expression originating from mitochondrial genes, expressed fewer than 200 distinct genes, and contained fewer than 500 UMIs (fig. S7C). We used less stringent thresholds for CEL-Seq data than for 10x data due to data quality. Data were normalized by sequencing depth, scaled to 10,000 counts, and log-transformed, and genes were scaled to have a mean of 0 and an SD of 1 using the Seurat NormalizeData function. Principal components analysis was performed on the scaled data using the 2000 most variable genes. Using 50 first principal components, we calculated a UMAP (*34*) two-dimensional representation of the data for visualization and calculated clusters using a community finding algorithm with resolution parameter set to 1. We performed these steps using the Seurat package in R (*30*, *31*).

### Cluster annotation

Using well-established marker genes of different cell types curated from the literature (table S2), we assigned a cell type to each cluster. Where two clusters were annotated as the same cell type, we merged them together. As further confirmation of our annotation, we next identified marker genes for each population algorithmically (table S6). To do this, we used a method that uses the tf-idf metric to identify genes specific to each population, as implemented in the “quickMarkers” function in the SoupX R package (*29*). We further filtered genes to include only those genes with a *P* value less than 0.01 after multiple hypothesis correction (hypergeometric test).

### Comparison of mouse and human adrenal medulla

Using an established map of the mouse medulla as our gold standard (*8*), we mapped this reference to our human data in two orthogonal ways. For this comparison, we restricted both human and mouse transcriptomes to those genes that could be unambiguously mapped between species (one-to-one orthologs). Using data from mice taken from embryonic day (E) 12.5 and E13.5 as independent references, we calculated a similarity score using logistic regression (*6*) for each human medulla cell for each of the four murine medullary populations. In addition, we used a canonical correlation analysis–based method to transfer the murine labels to the human medulla, as implemented in the Seurat R package (*30*). We identified differentially expressed genes between matching mouse and human medulla using edgeR (*35*) as described below. To obtain a list of genes with a large effect size difference between mouse and human, we filtered this list to include only genes with a false discovery rate (FDR) less than 0.01 absolute log fold change greater than 1, fraction of cells expressed greater than 80% in the species with highest expression, and less than 1% in the species with lowest expression. We further removed any gene that showed a significant difference between species in all four medullary populations (table S10).

### Differential expression in early and late medulla

To identify genes that were differentially expressed between early (<21 weeks) and late (>21 weeks) cell types in the adrenal medulla, we performed a differential gene expression analysis between early and late cells using FindMarkers function in Seurat for each medullary cell type (SCPs, bridge, sympathoblast, and chromaffin cells). We prefiltered genes that were detected at <25% frequency in either population. We identified all genes that passed the 0.01 *P* value threshold after multiple hypothesis correction (table S3).

### Differential expression in high-risk cancer cells with low and high sympathoblast signal

We categorized high-risk tumor cells as “low sympathoblast signal” and “high sympathoblast signal” by applying *k*-means clustering with *k* = 2 to the sympathoblast similarity score (logistic regression). To identify genes that were differentially expressed between low and high sympathoblast signal in high-risk cancer cells, we performed a differential gene expression analysis between low sympathoblast signal and high sympathoblast signal cells using FindMarkers function in Seurat. We prefiltered genes that were detected at <25% frequency in either population. We identified all genes that passed the *P* value threshold of 0.01 after multiple hypothesis correction (table S7).

### Pseudotime analysis

We ordered cells into a trajectory based on the similarity of their transcriptomes to create a pseudotime ordering of the medulla using monocle3 (*36*–*38*). We constructed a monocle3 object using data extracted from a Seurat object rather than reprocessing the data in monocle3. We used the “learn_graph” function to build a principal graph of the data. We then used “order_cells” function to place cells on a pseudotime trajectory, selecting a node (as defined in learn_graph) as a starting point of the trajectory. We used an SCP node based on previous knowledge from murine data that medullary cells originate from SCPs (*8*). In addition, to further investigate the identity spectrum between bridge and sympathoblasts, we performed the same analysis after subsetting the Seurat object to only include bridge cells and sympathoblasts.

### Differential expression of genes along pseudotime trajectory

To identify transcription factors that were differentially expressed along the graph of the medulla, we performed Moran’s *I* test using “graph_test” function in monocle3. We limited our gene set to the 1665 transcription factors (*37*) and identified all genes that passed the *P* value threshold of 0.001 after multiple hypothesis correction (table S11). In addition, to further investigate the identity spectrum between bridge and sympathoblasts, we performed the same analysis, this time using all genes, on just the bridge cells and sympathoblasts and identified all genes that passed the *P* value threshold of 0.001 after multiple hypothesis correction (table S11).

### RNA velocity

RNA velocity was calculated on the fetal adrenal reference map data using velocyto (*39*) to calculate the number of reads supporting spliced/unspliced isoforms of each gene in each cell. The velocity field projection to the UMAP embedding was then calculated using the velocyto.R R package, pooling 20 *k*-nearest neighbors and performing a gamma fit to the top/bottom 2% expression quantiles.

### Cell similarity calculation

To measure the similarity of a target single-cell transcriptome to a reference single-cell dataset, we used the methodology based on logistic regression outlined in detail in (*6*). Briefly, we trained a logistic regression model with elastic net regularization (α = 0.99) on the reference training set. We then used this trained model to infer a similarity score for each cell in the query dataset for each cell type in the reference data.

Softmax normalization was not used to allow for the possibility that some cells in the query dataset do not resemble any of the cell types in the reference dataset. Predicted logits were averaged within each cluster in the query dataset. This approach was implemented using the “glmnet” package in R (*40*).

### Genotyping individual cells in single-cell RNA-seq data

The details of the genotyping of individual cells are explained in detail in the Supplementary Materials with a brief description provided here. For each sample, regions of clonal copy number change and heterozygous SNPs were identified from either whole-genome sequencing or copy number arrays (table S4). Taking copy number regions resulting in allelic imbalance, heterozygous SNPs were phased together across the entire copy number aberration. In each single-cell transcriptome, the number of counts derived from either the major or minor allele was calculated for each copy number change by investigating the nucleotide sequence of the mRNA reads allele counter (https://github.com/cancerit/alleleCount) (*41*). For each cell, a posterior probability was calculated for both a diploid genome and the tumor genome. Figure 2 (C and D) shows the posterior probabilities of the tumor genotype for each cell. For downstream analysis, we considered only clusters of cells that had more cells that were definitively tumor (posterior probability >99%) than definitively normal (posterior probability <1%).

### Identification of cell type–specific genes

To identify genes that were specific to one cell type and no other in the adrenal medulla, we regenerated algorithmically defined markers as above, but with podocyte cells (*6*) included as a negative control. To use only the most specific markers, we applied a tfidf cutoff of 1 to select genes that were specific to each medullary cell type relative to all other cells in the dataset. Note that a tf-idf cutoff of *t* implies that the global rate at which a gene occurs in cells is less than exp(−*t*/rate_local), where rate_local is the rate at which the gene occurs in the cells in the target cluster.

We also removed genes that were expressed in more than 20% of cells in any other single cluster in the dataset. The resulting gene list consisted of genes specific to each cell type in developing adrenal medulla (“stringent_markers” category in the “dataset” column; table S6).

### Adrenal gland cell type signal in bulk RNA-seq data

To identify which adrenal cell type bulk RNA-seq neuroblastomas resembled most, we measured the expression levels of genes defined in table S6 in two bulk RNA-seq neuroblastoma datasets, TARGET (*14*) and SEQC (*15*). For both datasets, the transcripts per million reads (TPM) value was calculated for each gene in each sample. We defined a gene as being “present” in a sample when its log_2_(TPM) expression value exceeded that of the peak of the log_2_(TPM) distribution across all genes across all samples. For each gene, we then calculated the fraction of samples in which it was present, both globally and stratified by risk group.

### Defining neuroblastoma samples outside the adrenal gland

To define which neuroblastoma samples in TARGET (*14*) arose outside the adrenal gland, we filtered International Classification of Diseases for Oncology (*42*) description of each tumor by sites of occurrence. We excluded all samples where descriptions were absent or contained words “kidney,” “adrenal,” “abdominal,” “abdomen,” “unknown,” “retroperitoneum,” and “other,” identifying 21 samples where tumors occurred outside the adrenal gland.

### Differential expression between fetal adrenal medulla and tumor cells

To identify the key transcriptomic differences between neuroblastoma tumor cells and the normal fetal medulla, we performed differential expression analysis between fetal medulla single cells and tumor cells from CEL-Seq2 and 10x tumor datasets. For each dataset, we extracted tumor clusters and merged them with fetal adrenal medullary cells. We labeled all tumor cells as “tumor” and used the quickMarkers function from SoupX R package (*29*) to identify tumor-specific genes for each dataset. We used a cutoff of 0.01 *P* value after multiple hypothesis correction (hypergeometric test) and genes that were expressed in more than 20% of cells in any other single cluster in the dataset to define a set of tumor markers.

We merged the two marker lists and calculated an average tf-idf value for each gene present in either or both lists and used a tf-idf cutoff of 0.85 to define a single list of tumor markers. We then filtered the list to exclude genes expressed by more than 25% of leukocytes in each tumor dataset (table S9).

### Differential expression between low- and high-risk neuroblastomas in TARGET

To test whether any of the genes defined in table S9 displayed risk group dependence, we compared their expression levels between 4S and high-risk bulk neuroblastomas in the TARGET data (*14*). To do this, we used a negative binomial model, where we set the square root of the overdispersion value to 0.4 (as genuine biological replicates were not available). We constructed a generalized linear model, with age, MYCN status, and risk group as covariates and then used a quasi-likelihood *F* test to calculate genes for which the risk group coefficient was significantly nonzero [FDR, 0.05; edgeR (*35*) package] (table S12). We did not perform equivalent analysis in SEQC data, as the raw counts were not available for this dataset.

### Identification of neuroblastoma transcripts not expressed in postnatal tissues

To identify transcripts that were shared between cancer cells and the fetal medulla, we used the quickMarkers function in the SoupX R package to find the markers of cancer cells in each tumor dataset (CEL-seq and 10x). We removed genes that were expressed in fewer than 50% of cancer cells and genes that were expressed in more than 20% of cells in any other single cluster each dataset. We overlapped the gene lists for the two tumor datasets and filtered the list to exclude genes that were expressed in fewer than 10% of adrenal medullary cells in our reference. We then looked at the average expression [log_2_(TPM)] of those genes (table S8) in low- and high-risk neuroblastoma bulk samples and GTEx bulk samples (*21*). We sorted the gene list by increasing average expression outside the brain in GTEX data. The top 25 genes (i.e., genes with lowest expression outside the brain) are shown in Fig. 4A.

### Association between recurrent copy number changes and sympathoblastic expression

To compare regions of recurrent copy number changes in neuroblastoma to the genomic pattern of expression in reference cell populations, we first defined breakpoint regions and recurrently changed regions. We defined a recurrently changed region as one which had a gain (or a loss) in 200 or more samples (of 556) (*18*). For regions with a well-defined transition region (e.g., chromosome 11 change between recurrent gain and loss), we defined the breakpoint region to be 2 Mb around the transition point.

For sympathoblasts, erythrocytes, and chromaffin cells, we then calculated the average expression as a function of genomic position. To do this, we calculated Gaussian kernel smoothed density with bandwidth of 200,000 bp and weights equal to the library size normalized expression in each cell type placed at the transcription start site of each gene. This is roughly equivalent to a running mean of normalized expression of each cell type calculated across the genome.

This normalized, smoothed expression is what is plotted in Fig. 3F and fig. S8. To calculate the significance of the relative abundance of the sympathoblastic and chromaffin cells, we calculate the average difference in the smoothed expression between the two cell types in the regions of interest. To calculate a *P* value for the significance of the observed difference in smoothed expression, we compared the observed value to the value in 1000 randomly selected regions of the genome of the same size. We then defined the *P* value to be the fraction of the randomly selected regions with a difference greater than or equal to the observed one (fig. S5, B and C).

### Differential expression of cells carrying prognostic copy number change

In our 10x data, we identified one sample (PD43255) that had loss of both chromosome 1p and 11q, which was not present in any of the other tumor samples. To determine the transcriptomic consequences of these prognostically important copy number changes, we calculated the differentially expressed genes between those cells with and without the copy number changes. We limited this analysis to only those cells in the G_1_ phase of the cell cycle to exclude confounding with differences in proliferation. We then performed a quasi-likelihood *F* test using the differential expression testing package edgeR (*35*) comparing the cells with/without the copy number change. This test was performed with default parameters except for estimating the overdispersion parameter of the negative binomial distribution where we set prior.df = 0 to prevent information sharing between genes. This was done as there were sufficiently many cells in each group to robustly estimate genewise overdispersion without sharing information.

To test whether this list of differentially expressed genes related to sympathoblastic cells, we identified all markers of sympathoblastic genes with a tf-idf cutoff of 1 (*29*). We then performed a hypergeometric test to measure whether this list of sympathoblastic marker genes was overrepresented in the genes differentially expressed between tumor cells with and without the prognostic copy number changes.

### Risk group stratification for SEQC data

As COG (Children’s Oncology Group) risk group information was not available for the SEQC dataset, we stratified samples into risk groups based on *MYCN* amplification status, age at diagnosis, and disease stage. We defined samples as low risk if age at diagnosis was <18 months and *MYCN* amplification status was negative, excluding 4S stage samples. We defined samples as high risk if age at diagnosis was >18 months and *MYCN* amplification status was positive, excluding 4S stage samples.

### Code availability

We have included the source code used to generate the figures and tables presented in this analysis as data S1. The purpose of this code is to provide additional explanation of the analyses described in Materials and Methods, such as the precise function call and parameter values used.

## SUPPLEMENTARY MATERIALS

Supplementary material for this article is available at http://advances.sciencemag.org/cgi/content/full/7/6/eabd3311/DC1
